# Pancreatic Comorbidities in Pediatric Celiac Disease: Exocrine Pancreatic Insufficiency, Pancreatitis, and Diabetes Mellitus

**DOI:** 10.3390/diagnostics15101243

**Published:** 2025-05-14

**Authors:** Dimitri Poddighe

**Affiliations:** College of Health Sciences, Vin University, Gia Lam District, Hanoi 10000, Vietnam; dimitri.p@vinuni.edu.vn

**Keywords:** celiac disease, pancreatitis, diabetes, children, lipase, fecal elastase, exocrine pancreatic insufficiency

## Abstract

Celiac disease (CD) is a chronic and immune-mediated disorder triggered by the ingestion of gluten in some genetically predisposed individuals. CD can be associated with extra-gastrointestinal manifestations and diseases affecting several organs. In this review, the aim is to analyze and discuss the pancreatic alterations and/or comorbidities that could arise in the context of pediatric CD. Exocrine pancreatic insufficiency (EPI) can be observed in a variable fraction (up to 30%) of children diagnosed with CD at the diagnosis; indeed, it usually resolves after the implementation of a gluten-free diet (GFD). The main pathophysiological mechanisms of EPI could be represented by the impaired pattern of gastrointestinal hormones in CD patients. Conversely, pancreatitis seems to be a very rare comorbidity in CD children, since very few cases have been described in children. Therefore, there is no evidence that pancreatitis (including autoimmune forms) represents a relevant comorbidity in pediatric CD. Type 1 diabetes mellitus (T1DM) is a well-known and frequent comorbidity in CD children. The main determinant of this epidemiological association is the common HLA-related predisposing background, even if other (non-HLA-related) genetic and environmental factors (viruses, gut microbiome, and others) are likely to be also implicated in the development of both these autoimmune diseases. T1DM children with concomitant CD may experience specific challenges in the adherence to GFD, which has no negative impact on the glycemic and, in general, metabolic control of diabetes, if it is properly implemented and followed up.

## 1. Introduction

Celiac disease (CD) is a chronic and immune-mediated disorder triggered by the ingestion of gluten (a protein found in wheat, barley, rye, and other cereals) in some genetically predisposed individuals. In patients with CD, gluten intake elicits an abnormal immune response in the mucosa of the small intestine, causing lymphocyte infiltration and damage to the intestinal villi. Very briefly, this immuno-pathological process involves the activation of T-cells by gliadin-derived epitopes in subjects carrying specific HLA molecules (DQ2 and DQ8), which are expressed in nearly all individuals with CD and, basically, represent a necessary (but not sufficient) condition for the development of this disease. Accordingly, the main therapeutic resource in CD patients is represented by the implementation of a gluten-free diet (GFD) [1].

This immuno-pathological process in the intestinal mucosa results in malabsorption of nutrients and, thus, a variety of gastrointestinal symptoms; however, CD patients can also develop many different extra-intestinal manifestations, which may or may not be concomitant with the former ones [2]. However, whatever the clinical presentation of CD is, the (histopathological) hallmark of this disease is (a variable degree of) villous atrophy of the small bowel in the presence of mucosal infiltration by lymphocytes (intra-epithelial lymphocytes, IELs), as mentioned above. The Marsh classification describes the variable severity of the mucosal alterations in the duodenum of CD patients, which range from increased IELs (Marsh 1), passing through hyperplasia of the crypts with initial signs of villous damage (Marsh 2), to flattening of the villi (atrophy), which can be further described as partial (Marsh 3a), subtotal (Marsh 3b), and total (Marsh 3c) [3].

CD can occur and manifest at any age of life. The global prevalence of CD in children has been constantly increasing over the past few decades, partly due to greater disease awareness, improved diagnostic methods, and increased screening in at-risk populations. The current estimation is that at least 1 in 100 children worldwide is affected by this disease [4]. There is significant geographic variability in the incidence and prevalence of pediatric CD, due to both genetic differences (in terms of predisposing HLA haplotypes) and exposure to dietary gluten. Higher rates are observed in Europe and North America, where this observation is also due to greater disease awareness and diagnostic resources. Indeed, CD prevalence is much lower in other geographical areas, especially in Asia, although recent studies suggested that CD could be underdiagnosed in several regions of this continent and could increase in the near future, also considering the ongoing changes in dietary habits. Accordingly, in countries with higher rates of CD, it is possible to see an increased number of diagnoses in immigrant populations from countries characterized by a low CD prevalence [4,5,6,7,8].

As mentioned above, extra-gastrointestinal manifestations can be part of the clinical picture of CD and are not infrequent in pediatric patients, too. In CD children, the most frequent extra-gastrointestinal manifestations are related to nutritional deficiencies (e.g., anemia, enamel defects, aphthous disease, behavioral disturbances, etc.) and/or growth disturbances (short stature, delayed puberty, and low weight). Moreover, these patients can also present comorbidities related to a general autoimmune dysregulation and/or similar genetic background (e.g., type 1 diabetes mellitus, thyroiditis, autoimmune hepatitis, juvenile idiopathic arthritis, etc.). Therefore, many organs and systems can be affected in CD patients, including the pancreas [9,10].

The pancreas is a glandular organ with both exocrine and endocrine functions. Briefly, the exocrine component of the pancreas is composed of acinar cells and ductal cells. The acinar cells are the primary secretory units, which produce digestive enzymes, while the ductal cells can modify the composition of their secretion proceeding from the acini to progressively larger ducts, until being finally excreted into the duodenal lumen.

Pancreatic enzymes are important for the digestion of several nutrients, including carbohydrates (amylase), proteins (trypsin, chymotrypsin, elastase, and carboxypeptidases), lipids (lipase), and nucleic acids (ribonuclease and deoxyribonuclease). These enzymes are secreted as zymogens (inactive precursors) to prevent autodigestion of pancreatic tissue. Notably, during the transit in the pancreatic ductal system, a significant amount of bicarbonate is added to this pancreatic secretion through the exchange of chloride ions in a process mediated by the cystic fibrosis transmembrane conductance regulator (CFTR) and anion exchangers present in the membranes of ductal cells. Indeed, chyme entering the duodenum from the stomach is acid, and such a bicarbonate content of the pancreatic secretion can create a slightly alkaline environment in the duodenum, which is essential for the optimal activity of the pancreatic enzymes. This bicarbonate secretion is tightly regulated by hormonal (secretin and cholecystokinin) and neural (vagal stimulation) factors. This neuro-hormonal network also stimulates the enzymatic secretion by the acinar cells of the pancreas: in detail, secretin and cholecystokinin are, respectively, released in response to low pH and fat/protein presence in the duodenum, and both hormones are important for the activity of the exocrine pancreas [11,12,13].

The endocrine pancreas consists of specialized clusters of cells known as the islets of Langerhans, which can secrete several hormones and account for approximately 1–2% of the total pancreatic mass. These islets are scattered throughout the pancreas and contain several different cell types, and each one is responsible for secreting a specific hormone: (i) Alpha (α) cells (glucagon); (ii) Beta (β) cells (insulin); (iii) Delta (δ) cells (somatostatin); (iv) PP cells (pancreatic polypeptide); and (v) Epsilon (ε) cells (ghrelin) [14].

In this review, the aim is to analyze and discuss the pancreatic alterations and/or comorbidities that could arise in the context of pediatric CD.

## 2. Exocrine Pancreatic Insufficiency

Exocrine pancreatic insufficiency (EPI) refers to the inadequate secretion of digestive enzymes, which leads to malabsorption and gastrointestinal symptoms such as diarrhea, weight loss, and abdominal discomfort. These are all clinical manifestations that can be observed in CD patients, and, therefore, pancreatic dysfunction could represent an additional pathophysiological mechanism in this disease, beyond the general nutrient malabsorption due to villous atrophy and the consequent reduction in intestinal surface available to this specific aspect of the digestive process [15].

A recent position paper by the American Gastroenterological Association (AGA) stated that EPI should be considered in patients with “moderate-risk clinical conditions”, including CD, among others [16]. Moreover, the systematic review and meta-analysis by Jiang et al. highlighted a relatively high prevalence of EPI in adult patients diagnosed with CD. In their pool of newly diagnosed CD patients, the EPI prevalence was 26.2% (with a range of 10.5–46.5% across the selected studies); as regards CD patients on a GFD for at least 9 months, the pooled EPI prevalence was 8% (with a range of 1.9–18.2% according to the different studies) [17].

There is consistent evidence about the occurrence of EPI in CD children, too. The first reports regarding the co-existence of EPI in CD patients (including children and especially in the context of GFD treatment failure) date back to 1980 [18,19]. One of the first studies on this matter was published by Carroccio et al. in 1991. These authors investigated the occurrence and severity of EPI in untreated pediatric CD and after a GFD period, by using a secretin–cerulean test (which consists in measuring the duodenal concentration of bicarbonate and enzymatic activity of lipase, phospholipase, and chymotrypsin, after an intravenous injection of secretin and cerulean) and also by evaluating the fecal chymotrypsin concentration. Although no significant differences were observed in terms of duodenal output of pancreatic enzymes and bicarbonate among these CD groups and controls, 23% of untreated CD children showed trypsin and/or lipase activity below the normal range; moreover, the fecal chymotrypsin concentration was significantly lower in untreated CD children than in GFD-treated ones and controls [20].

The same authors eventually confirmed that approximately 30% of CD children can develop EPI to some extent. In this study, the new information was that EPI did not correlate with the impairment of patients’ nutritional status and serum albumin levels in most cases [21]. They also validated the use of another test, namely fecal elastase, as a diagnostic tool to assess the pancreatic impairment in CD patients, in addition to fecal chymotrypsin or other assays previously described [22,23]. Other researchers showed that fecal elastase levels can be reduced in CD children and normalize after GFD. However, it is clear that EPI is not a constant alteration in CD children at the diagnosis, even if the intestinal mucosa is atrophic [24,25,26,27].

Other pediatric studies investigated the blood level of pancreatic enzymes in the context of (potential) EPI in pediatric CD. Nousia-Arvanitakis et al. measured serum amylase (in addition to fecal elastase) in 36 CD children and adolescents, before and after GFD. Briefly, untreated CD patients (with villous atrophy) showed reduced serum amylases compared to both patients with normalized mucosal histology after GFD and matched healthy controls. Serum pancreatic amylase activity increased to normal values after GFD, and fecal elastase activity decreased to normal values (below 200 μg/g of stool) under the same conditions. Notably, these alterations in pancreatic enzymes inversely correlated with the degree of intestinal damage; moreover, the ultrasound findings of the pancreas were normal, regardless of the enzyme activity or mucosal histology [28]. Later, the same authors published another pediatric study demonstrating that plasma cholecystokinin (CCK) release in response to oral nutrients was impaired in CD children with duodenal mucosal atrophy, which could cause reduced stimulation of exocrine pancreatic activity during the digestive process [29].

All these studies suggested that EPI is not uncommon in untreated CD children and is reversible after GFD. Therefore, replacement therapy with pancreatic enzymes is not necessary in general, even if it may be considered in some CD children in the first month after diagnosis to promote a greater weight recovery [30]. Indeed, some authors have considered the therapy with pancreatic enzyme supplements in adults with persisting symptoms despite 6–12 months of a well-implemented GFD and, in general, non-responsive CD [31]. However, one trial (NCT02475369) was recently carried out in patients >18 years with GFD-unresponsive CD, and it did not show any significant symptoms improvement in these patients [32].

In summary, even though the pathophysiology of EPI in CD is not fully elucidated and could be multifactorial, the available studies suggest that the impaired secretion of pancreatic stimulating hormones (due to atrophic alterations of the intestinal mucosa), such as CCK and secretin, could be the main pathophysiological mechanism explaining this pancreatic comorbidity; indeed, no structural alterations can be detected in the pancreas of CD patients developing EPI [33,34]. Notably, this hypothesis is also consistent with other extra-intestinal alterations that are often observed in CD patients, like gallbladder dysmotility and some perturbations of bile composition, which also reflect an impaired pattern and/or amount of CCK secretion by the specific duodenal enteroendocrine cells during the digestive phase [35,36].

## 3. Pancreatitis

The term “pancreatitis” generally refers to the inflammation of the pancreas. Clinically, it is characterized by symptoms such as abdominal pain, nausea, and vomiting, which are usually associated with elevated levels of pancreatic enzymes like amylase and lipase in the bloodstream. Schematically, pancreatitis can be classified into acute, chronic, or recurrent types, based on the frequency and duration of episodes [37]. 

Pancreatitis is not a common disease in the pediatric population. The incidence of acute pancreatitis (AP) in the pediatric literature is estimated to be <15/100,000 children, even though the rates of diagnosis are rising [38,39]. In general, AP is diagnosed based on the following aspects: suggestive (acute) abdominal pain, increase in serum amylase and/or lipase, and imaging consistent with pancreatic inflammation [37,40].

Acute recurrent pancreatitis (ARP) can be defined as the occurrence of at least two episodes of AP, with either complete resolution of pain (>1 month) or complete normalization of serum pancreatic enzymes between these episodes. It usually affects around 25% of children with a previous episode of AP [37].

Chronic pancreatitis (CP) is characterized by persistent pathological changes in the pancreatic parenchyma (e.g., fibrosis, calcification, and glandular atrophy) and pancreatic ductal abnormalities (e.g., intraductal calculi, stenosis and strictures, and peri-pancreatic fluid collections), which can be demonstrated by different types of radiological imaging. Children with CP can variably present steatorrhea and/or malodorous stools, bloating, and abdominal pain, in addition to general malnutrition and/or fat-soluble vitamin deficiency [41,42].

A very short but specific mention should be given to autoimmune pancreatitis (AIP), considering the autoimmunity context of CD. AIP represents <5–10% of pancreatitis cases (prevalence of 1–2/100,000 persons) and even less in children. Based on histopathological criteria, AIP is classified into two main subtypes that are mainly distinguished by the absence (type 1 AIP) or presence (type 2 AIP) of one peculiar finding, called as “granulocytic epithelial lesion” (GEL), consisting of the neutrophilic infiltration of medium/small-sized ducts and often acini. Both AIP types are almost exclusively adulthood diseases; however, AIP has been rarely described in the pediatric population, where these few reported cases are consistent with type 2 AIP in most cases [43,44,45,46].

As described in the previous section, several studies have indicated an association between CD and pancreatic dysfunction in some patients. However, the association between CD and “clinically evident” pancreatitis in children is not so well established. According to a study by Carroccio et al., around 25% of CD adult and pediatric patients displayed elevated pancreatic enzyme values (lipase and amylase), which are commonly used to diagnose pancreatitis. However, few patients had a history of (recurrent) abdominal pain consistent with pancreatitis: therefore, these abnormalities were found in CD patients without clear manifestations or even completely asymptomatic [47]. In the study by Migliori et al., which also included some children (age range: 8–69 years), 65 subjects with a “benign” increase in pancreatic enzymes were screened for CD. Only one patient was diagnosed with CD and, according to these authors, this enzymatic increase could not be specifically attributed to CD [48].

The first and largest study assessing the occurrence of pancreatitis in CD patients (in general) was authored by Ludvigsson et al. This was a population-based study, which found a positive association between CD and pancreatitis. Notably, this study mainly included young patients and, according to the demographic information provided in the paper, most patients were of pediatric age at the study entry. Around 74% of the study participants (10,573 individuals with CD and 51,283 reference individuals) were 16 years or older at the end of follow-up. However, although CD was associated with a 3-fold-higher risk of pancreatitis, such a risk increase for any form of pancreatitis was only seen in individuals with CD diagnosed in adulthood. The authors acknowledged that the insufficient follow-up time may explain why this study was not able to highlight the risk of subsequent pancreatitis among individuals with CD diagnosed in childhood [49]. Similar conclusions have emerged from other and more recent population-based studies from Sweden again [50], but also from other geographical areas [51,52]. For instance, Alkhayyat et al. in the US found that CD patients were at increased risk of developing AP and CP. These authors also suggested that clinical conditions of recurrent abdominal pain/idiopathic pancreatitis and the elevation of pancreatic enzymes should lead to a consideration of CD screening. Even though 5.6% of the CD population was represented by children and adolescents, unfortunately no specific age-related analysis or results are provided in this article [51].

The first well-defined case of AP in a CD child that we could retrieve in our literature research was described by Bultron et al. in 2009. This was a 9-year-old boy with a previous medical history consistent with CD, who complained of severe abdominal pain and vomiting. He displayed increased serum amylase and lipase levels. Both abdominal ultrasound and computed tomography demonstrated a diffusely edematous pancreas, but no common bile duct dilation, stones, or sludge were present. No family history or other risk factors for acute (recurrent) pancreatitis could be highlighted. At that time, only a limited number of adult CD patients had been previously reported as affected by acute or chronic pancreatitis [53].

Eventually, only three case reports described the association of CD and pancreatitis in children [54,55,56]. Moreover, these clinical descriptions present some informative gaps and/or inconsistencies, especially as regards the diagnosis of CD, as summarized in Table 1. For instance, Halabi et al. reported a negative CD serology, even if the duodenal histology showed total villous atrophy; the diagnosis of CD was assumed from the histological response to a 6-month GFD. Conversely, like the first case report by Bultron et al. [53], the most recent case report by Patel et al. had no clear information about duodenal histopathological findings; notably, this latter case of pancreatitis was finally diagnosed as type 2 AIP [55]. Finally, Sultan et al. described a patient whose diagnosis of CD was confirmed by both serology and histopathology [56].

Interestingly, a recent study analyzed the clinical characteristics of pancreatitis in pre-adolescent children. During the 10-year study period, the authors described 85 pediatric patients diagnosed with AP: notably, 3 of these cases were diagnosed with CD [57].

Despite such very limited evidence about the occurrence of pancreatitis in CD children, some experts have agreed that CD should be screened in some patients and circumstances [58]. However, according to the present literature analysis, we can conclude that there is no evidence that CD children are at higher risk of pancreatitis, even though this risk profile seems to be different in adult patients, perhaps due to the concomitance of other factors promoting pancreatic injury.

## 4. Type 1 Diabetes Mellitus

The epidemiological association between CD and type 1 diabetes mellitus (T1DM) is well known. A systematic review highlighted that the coexistence of these diseases in children can be variable according to different studies and populations: based on serological testing, the prevalence of CD in children with T1DM varies between 1.4% and 24.5%, and T1DM children with biopsy-confirmed CD are between 1.1% and 16.6% [59]. A recent and large nationwide cohort study from Sweden (including >90% of all the pediatric patients diagnosed in the country during the 7-year study period) showed a 9.8% prevalence of CD in children with T1DM. Notably, >50% of the study participants with this disease association received the diagnosis of CD before or at the onset of T1DM, whereas most of the remaining patients were diagnosed with CD within 5 years after T1DM onset. In summary, >95% of diabetic children with concomitant CD developed this comorbidity before or within a 5-year period after the diagnosis of T1DM [60].

To our knowledge, the first report about the comorbidity between CD and T1DM dates back to 1969, when an 8-year-old child admitted with a 2-week history of polyuria and polydipsia was diagnosed with T1DM; however, due to a clinical history of intermittent diarrhea for several years, he underwent a small-bowel biopsy, which revealed a flat mucosa consistent with CD. Finally, GFD led to an immediate clinical improvement with weight gain [61]. These authors described this case following an article by Hooft et al. about the association between diabetes and malabsorption in some children with steatorrhea [62]. Immediately after this first report by Walker-Smith et al., several authors described similar cases from different countries (UK, Finland, Belgium, Canada, and Australia), which suggested that malabsorption and, in detail, CD in diabetic children was not only a coincidence [63,64,65,66,67].

In 1974, Thain et al. reported that the “HLA-8 and HLA-1 antigens” were more frequent in CD in patients and their families, which was suggested to predispose people to this disease [68]. This hypothesis was eventually confirmed by several studies [69,70,71]. Nowadays, it is well known that a major role in the genetic predisposition to both CD and T1DM and, consequently, their epidemiological association, is played by the common HLA background, namely HLA-DR3-DQ2 (HLA-DRB1*04-DQA1*03:01-DQB1*03:02) and HLA-DR4-DQ8 (HLA-DRB1*03-DQA1*05:01-DQB1*02:01) haplotypes, which are considered to account for approximately 50% of the overall genetic risk in both these diseases. Whereas the highest-risk genotype for CD is represented by the HLA-DQ2 homozygosity, the double heterozygosity for HLA-DQ2 and HLA-DQ8 is associated with the highest risk for T1DM [72,73]. A recent systematic review further highlighted the prominent role of the DQ8 heterodimer in CD patients who are also affected by T1DM [74], as previously suggested by some specific studies from different geographical regions [75,76,77].

However, these HLA-DQ variants are relatively common in the general population, and most carrier people neither develop CD nor T1DM during their lifetime [78,79]. Therefore, other genetic factors (not HLA-related) are definitely implicated in the etiology of both CD and T1DM and, probably, in their “preferential” association [80,81]. The study by Smyth DJ et al. showed that 21 non-HLA loci were associated with T1DM and 11 non-HLA loci were associated with CD: interestingly, 3 CD loci resulted to have an association with T1DM (RGS1, TAGAP, and IL18RAP) and T1DM loci were also associated with some CD loci (PTPN2 and CCR5) [82]. Other research has supported this concept, and several genes have been implicated according to different studies [83,84]. Very recently, Kaur et al. suggested that non-HLA polymorphisms in some genes, such as PTPN22 and INS, could provide further and specific susceptibility to develop the association T1DM + CD, rather than the isolated form of T1DM. Moreover, they also showed that immunodominant epitopes of some autoantigens of each respective disease share 40% homology [85]

Indeed, the cross-reaction and epitope spreading between these autoantigens could provide an additional mechanism for this comorbidity. Both T1DM and CD are characterized by the production of specific autoantibodies. As mentioned, anti-tissue transglutaminase (anti-tTG) antibodies are the main serological marker of CD. As regards T1DM, insulin autoantibodies, islet cell antibodies, glutamic acid decarboxylase autoantibodies, insulinoma-associated antigen-2 autoantibodies, and autoantibodies against zinc transporter T8 can be detected. Notably, whereas anti-tTG antibodies have a >90% positive predictive value for CD, this is not the case for the aforementioned T1DM-related autoantibodies: indeed, the positive predictive value for T1DM of a single autoantibody is usually only around 10–25% and can increase up to 75% in case of positivity of three or more autoantibodies [86,87,88].

However, the interplay between autoantibodies of the two diseases cannot be limited to some autoantigens’ homology and, then, cross-reaction. Some authors suggested that tTG could even be expressed in the pancreatic islets in some specific and pathological circumstances, especially under stress and/or inflammatory conditions, as may happen in children with T1DM. Such an expression of CD-related autoantigens in the pancreas could further trigger immunological co-sensitization in children with T1DM and, then, promote the concomitant occurrence of CD in this pathological context, as proposed by some authors [89,90,91]. The observation that T1DM autoantibodies precede anti-tTG autoantibodies more often (67%) than vice versa (27%), as evidenced by the TEDDY (The Environmental Determinants of Diabetes in the Young) prospective birth cohort study (which included children at high HLA genetic risk of both T1DM and CD), could be consistent with the aforementioned hypothesis and, accordingly, a preexisting T1DM autoimmune background may further promote the development of CD autoimmunity [92]. Of course, in addition to genetics, environmental or epigenetic factors should be implicated in the pathogenesis of both disorders and could even play a role in their association [93]. Among these, viral infections and gut microbes are two players that have attracted most attention on this matter.

Some peculiarities of the gut microbiome have been highlighted for both CD and T1DM: interestingly, fewer *Firmicutes* spp. and more *Bacteroides* spp. in the gut have been observed in both diseases according to some studies, even if they are not all concordant [94,95,96,97]. Then, some authors proposed that the intestinal microbiome may develop in a similar way in both diseases [92]. The composition of the gut microbiome in children predisposed to and affected by CD and/or T1DM could be in part related to the HLA genetic background, since HLA-DQ2 and -DQ8 might favor some microbiological groups compared to others in the complex, and bidirectional interaction between gut microbiota and host immune system [98,99]. Gluten might also be implicated to some extent: some authors have proposed that gluten could cause an inflammatory-mediated increase in mucosal permeability in the gut and/or impact the composition of the intestinal flora, but this hypothesis mainly derives from animal studies [100,101,102]. Conversely, the gut microbiota could also influence the antigenic effect of some nutrients (including those related to gluten) and, thus, their impact on the development of autoimmunity, including the concomitant occurrence of T1DM and CD, specifically [103,104]. However, the available studies cannot lead to any mechanistic conclusion or evidence about any specific microbiome signature and/or markers of progression to T1DM, CD, or both diseases [105].

As mentioned, the role of gastrointestinal infections and, in detail, viruses, has also been considered in such a disease association, even if there is no final evidence on this point either [93,106,107]. Notably, some findings from the TEDDY study could be consistent with this hypothesis, like the fact that a gastrointestinal infection was significantly associated with anti-tTG seroconversion a few months later in this cohort of patients at high risk for T1DM [92,108]. A potential relationship between rotavirus and autoimmunity has been raised for both T1DM and CD by some studies, but no conclusive evidence has been achieved [92,109,110]. Some data analyses from the TEDDY study may suggest a protective role of rotavirus vaccination under specific circumstances, such as gluten exposure before 6 months of age [108,111]. However, in general, most more recent studies are not consistent with this aspect [112,113,114,115]. Among them, a recent and large cohort study from the UK by Inns et al. investigated the pediatric rotavirus vaccination from the perspective of a potential risk for both T1DM and CD: no evidence came up that children vaccinated against rotavirus can have a lower incidence of CD and T1DM than those without this vaccine [115].

From the clinical point of view, it is worth spending some words on the impact of GFD in children developing both CD and T1DM, since some studies have tried to address this issue. Goh et al. did not observe any difference in HbA1c levels after a 1-year GFD between T1DM children with asymptomatic CD (diagnosed through targeted screening) and T1DM children without CD [116]. Actually, some authors found that the diagnosis of CD and the consequent implementation of GFD could be associated with an increase in HbA1c during the first year of this dietary regimen, compared to T1DM children without concomitant CD [115]. However, the most recent studies [117,118,119,120,121] and literature analyses [122,123] do not support any detrimental effect of GFD in T1DM children diagnosed with CD. Among the latter ones, the systematic literature review and meta-analysis by Mozzillo et al. showed no significant differences between T1DM children and young patients with and without CD (and, then, with and without GFD, respectively) as regards the diabetic control in terms of HbA1c levels, number of hypoglycemic episodes, and insulin requirements [122]. Eland et al. also concluded that GFD in pediatric patients with T1DM and CD can be implemented without any negative effect on HbA1c or insulin therapy [120]. Some studies have variably investigated the potential benefit of GFD in children affected with both T1DM and CD on their metabolic control (glycemic values and/or lipid profiles) and growth parameters [123,124,125,126]. Interestingly, a recent study also speculated that GFD in recent-onset T1DM children without concomitant CD might be associated with a slower decline in β-cell function and better metabolic control and HbA1c [127], but no further studies in this direction have been published in the last few years. 

Finally, it is important to highlight that the implementation of and adherence to GFD could pose additional challenges to T1DM children diagnosed with CD. Recently, Soderstrom et al. analyzed the compliance to a GFD in this pediatric population: these children showed a lower level of GFD adherence to some extent (compared to children with isolated CD), and this situation was more prevalent in older (T1DM + CD) children with poorer metabolic control [128]. Similar findings were observed by Verma A et al. in India, who reported that only 33.3% of children with both diseases were fully adherent to GFD compared to 56.3% of children with isolated CD [129]. These studies indicate that children with this comorbidity could need intensified dietary support and primary care follow-up in order to increase their awareness about the importance of GFD for CD treatment, especially for the most vulnerable subjects and in resource-limited social and medical settings [128,129,130].

## 5. Other Potential Pancreatic Comorbidities?

Recently, Pes et al. explored the potential relationship between CD and (gastro-entero-)pancreatic neuroendocrine neoplasms [131]. However, among the 19 articles included in their literature research, only 3 cases of pancreatic neuroendocrine neoplasms were retrieved, which were of different types (somatostatinoma, VIPoma, and neuroendocrine carcinoma) and were all diagnosed in adult patients [132,133,134]. Therefore, even if further analyses could be required to better elucidate any potential link between CD and gastro-entero-pancreatic neuroendocrine neoplasms in general, there is no current evidence that pancreatic neuroendocrine tumors could be a relevant comorbidity of pediatric CD. Moreover, in the rare occurrence of gastro-entero-pancreatic neuroendocrine tumors in children, the tumor site was always limited to the gastrointestinal tract [135,136].

As regards pancreatic cancer, even if there are some studies investigating the potential association with CD, these are all related to adult patients [137,138,139]. Indeed, pediatric pancreatic neoplasms are extremely rare and usually benign [140]. Moreover, (autoimmune) pancreatitis (whose potential role as a facilitating factor for pancreatic neoplasms has been considered in some articles) [141,142,143] is not associated with CD in children, as discussed above. Therefore, the risk of pediatric cancer is very low in general [144], and, in detail, there are no cases described in the context of pediatric CD.

## 6. Conclusions

**Figure 1:**
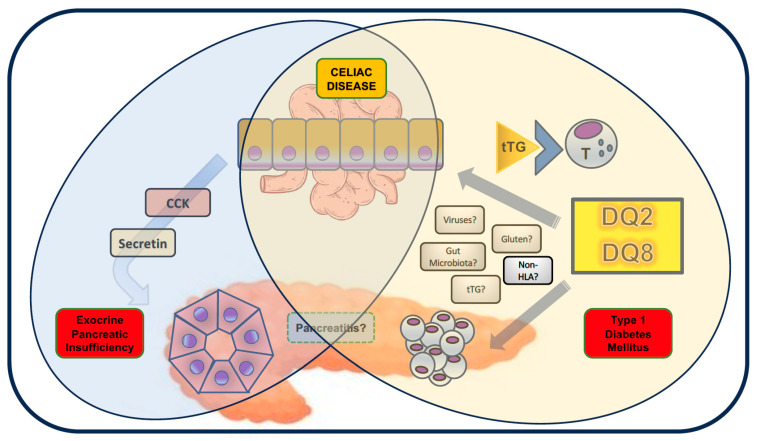
Graphical overview of pancreatic comorbidities in CD children. EPI is caused by the altered secretory pattern of gastrointestinal hormones (in particular, cholecystokinin and secretin) secreted by the intestinal mucosa, which is atrophic in these patients. The association of DMT1 and CD is partially explained by the common background of HLA predisposition, although other genetic (non-HLA) and environmental factors (gut microbiome, viral infection, etc.) are necessary for the occurrence of both diseases and may also influence their association. Gluten exposure may support the occurrence of DMT1 by altering intestinal permeability and/or promoting an inflammatory environment, favoring the development of autoimmunity; beta-cells may also express tTG antigens under conditions of cellular stress and injury. The association between CD and any form of pancreatitis is not supported by any significant evidence. Some items of this figure were obtained from https://www.freepik.com (accessed on 16 April 2025).

The present literature review analyzed the current evidence on pancreatic comorbidities (EPI, pancreatitis, and T1DM) in the pediatric population, as graphically summarized in Figure 1.

EPI can be observed in a variable fraction (up to 30%) of children diagnosed with CD; however, it represents a transient problem, since it usually resolves within 6–12 months after the implementation of GFD. This finding is consistent with the fact that the main pathophysiologic mechanisms of EPI in CD children could be represented by the impaired pattern of gastrointestinal hormones secreted by the enteroendocrine cells of the atrophic intestinal mucosa in these patients. Indeed, replacement therapy with pancreatic enzymes is not indicated, except in a few specific cases showing severe EPI-related gastrointestinal manifestations and/or growth/nutritional deficits during the first months after CD diagnosis.

Conversely, pancreatitis seems to be a very rare comorbidity in CD children. Indeed, very few cases were described in children and only one was represented by AIP. Therefore, there is no evidence that pancreatitis (in any form) represents a relevant comorbidity in pediatric CD.

T1DM is a well-known and frequent comorbidity in CD children. CD is diagnosed in around 5–10% of T1DM children, even if there is a variability among different studies (1–16%). The main determinant of this epidemiological association could be the common HLA-related predisposing background, even if other (non-HLA-related) genetic and environmental factors (viruses, gut microbiome, gluten exposure, and others) are probably necessary for the development of both these autoimmune diseases. Although GFD can include foods with a high glycemic index, most studies showed that it does not have any detrimental effect on the metabolic control in T1DM children, if it is properly implemented and followed up. Indeed, compliance to a GFD in diabetic children diagnosed with CD could be more difficult, and they should have more primary care and dietary support to improve their adherence to GFD.

## Figures and Tables

**Table 1:** Specific diagnostic work-up in CD patients.

Article	Age Sex	Acute Symptoms	Timing (vs. CD Onset)	Amylase (U/L)	Lipase (U/L)	Anti-tTG IgA (U/mL)	Duodenal Biopsy	Follow-Up	Relapse	Notes
(Year)										
[Country]										
***Bultron*** (2009) [USA]	9 yrs. M	Emesis Abdominal Pain	diagnosis	351	1657	31	n/a	6 mo	N	-
***Halabi*** (2010) [USA]	3 yrs. M	Emesis Abdominal Pain	diagnosis	4513	2343	3	TVA	6 mo	N	CD
										serology negative
***Sultan*** (2015) [Palestine]	12 yrs. F	Emesis Abdominal Pain	diagnosis	470	n/a	55	TVA (Marsh III)	n/a	N	-
***Patel*** (2019) [Canada]	4 yrs. F	Abdominal Pain Jaundice	6–12 mo	n/a	H	19.4	n/a	18 mo	N	AIP
										Type 2

**Abbreviations:** TVA, total villous atrophy; N, no; H, high; n/a, not available; yrs., years; mo, months; M, male; F, female.

## Data Availability

Not applicable.
